# The Basis of Patient Resistance to Opportunistic Discussions About Weight in Primary Care

**DOI:** 10.1080/10410236.2023.2266622

**Published:** 2023-10-30

**Authors:** Madeleine Tremblett, Helena Webb, Sue Ziebland, Elizabeth Stokoe, Paul Aveyard, Charlotte Albury

**Affiliations:** a Nuffield Department of Primary Care Health Sciences, University of Oxford; b School of Social Sciences, University of the West of England; c School of Computer Science, University of Nottingham; d Department of Psychological and Behavioural Science, The London School of Economics and Political Science

## Abstract

Clinicians expect that talking to patients with obesity about potential/future weight loss will be a difficult conversation, especially if it is not the reason that a patient is seeking medical help. Despite this expectation, many governments ask clinicians to take every opportunity to talk to patients about weight to help manage increasing levels of obesity. Although this is recommended, little is known about what happens in consultations when clinicians opportunistically talk to patients about weight, and if the anticipated difficulties are reality. This paper examines displays of explicit patient resistance following opportunistic weight-loss conversations initiated by GPs. We analyzed audio recordings and transcribed them for conversation analysis. We focused on the precursors of explicit resistance displays during opportunistic weight loss discussions, the format of the resistance, and the ways it was managed by GPs. We found relatively few instances of explicit resistance displays. When it did occur, rather than be related to the opportunistic nature of the advice, or the topic of weight itself, resistance was nuanced and associated to the sensitivity of the GPs managing unknown patient levels of awareness of weight loss benefits, or prior efforts to lose weight. Clinicians tended not to challenge this resistance from patients, and we suggest this tactic may be acceptable to patients and help foster the long-term collaborative relationships needed to tackle obesity. Data are in British English.

## Introduction

Weight has been reported as a sensitive topic for patients living with obesity, as well as for clinicians. Patients report that conversations with clinicians that problematize their weight and broach weight loss can be upsetting and stigmatizing (Ananthakumar et al., 2020). Clinicians also report concerns about discussing weight with patients living with obesity (Warr et al., 2020) and often worry that patients may feel criticized (Blackburn & Stathi, 2019). Despite these concerns, conversations about weight can be experienced as helpful and motivational (Aveyard et al., 2016; Potter et al., 2001) and, when handled sensitively and in an understanding way, can be welcomed (Talbot et al., 2021).

Talk about weight often leads to discussions of health behaviors. Weight loss, through diet and physical activity, tends to be the first “treatment” option for patients living with obesity (e.g. (National Institute for Health and Care Excellence [NICE], 2022). Clinicians may suggest that patients change diet and physical activity to address obesity, based on assumptions (rather than knowledge) about patients’ current behaviors (Ananthakumar et al., 2020). This can lead to conversations that imply blame, or shame, and where patients may be advised to make changes they have already implemented. Patients report receiving this “banal” and “flippant” advice (Ananthakumar et al., 2020) negatively.

If weight can be a difficult topic to discuss in healthcare settings generally, introducing weight opportunistically could amplify the difficulties. In the United Kingdom (UK), primary care doctors (GPs) are recommended to use their clinical judgment on when to identify overweight or obesity, asking a patient’s permission to talk about their weight and discuss it sensitively, before discussing interventions (taking into account individual’s needs and preferences) (NICE, 2022), with an aim to offer support for weight loss to everyone with obesity (Department of Health and Social Care, 2020). Evidence shows that even very brief opportunistic weight loss advice is associated with patient weight loss (Aveyard et al., 2016). However, clinicians see weight loss as a low priority topic, citing concerns that they are unsure what to say, and fear causing offense (Warr et al., 2020). These concerns are typically reported in post-hoc reports of consultations (e.g., interviews or surveys) rather than via direct observations of consultations themselves (Warr et al., 2020).

There have been few examinations of patient responses to weight loss advice, except in the context of referrals to weight management support (Albury et al., 2020). We have previously outlined that opportunistic discussions about weight loss are responded to better when clinicians display delicacy in their approach (Tremblett et al., 2022). However, in this prior study, the focus was not on what happens when patients display explicit resistance during opportunistic weight loss advice and how that can be managed. This is important, as a barrier for clinicians to deliver opportunistic health behavior advice is the anticipated explicit resistance from patients, which could lead to an extension of consultation times (Hansson et al., 2011).

Patient resistance to clinical advice has been regularly observed in conversation analytic studies across a wide range of healthcare contexts. “Resistance” has been described empirically in terms of “resistance displays” and recently has been conceptualized as ‘participants (temporarily) suspending their cooperation in the joint “definition of the situation” (Goffman, 1959)’ (Huma et al., 2023, p. 501). Huma et al. (2023) state responses can resist some aspect of an initiating action, whilst aligning with others. Consequently, responding to advice with a dispreferred response may be one way of demonstrating resistance (e.g., Bloch & Antaki, 2022; Heritage & Sefi, 1992) but is not always a display of resistance (Huma et al., 2023). Resistance displays to talking about health behaviors – which could be a recommendation, advice or information (Pilnick, 1999) – occurs typically in the “treatment recommendation” slot of a consultation (Pilnick & Coleman, 2003; Sorjonen et al., 2006). Furthermore, resistance displays tend to follow generalized or unspecific recommendations (Koenig, 2011; Stivers, 2005), when a patient’s health behaviors were raised as a cause of illness (Albury et al., 2019), or when health behavior change talk was pursued by clinicians despite patients displaying they did not wish to discuss it (Albury et al., 2019).

Existing research in healthcare settings shows resistance displays are often passive, not explicit. Explicit resistance can be seen in conversations through responses that include refusals, rejections, disagreements, counters, and challenges (Joyce, 2022; Muntigl, 2013). In contrast, passive resistance, which may indicate “difficulty” by also having some implications for progressivity, includes silence, hesitation, unmarked acknowledgment (Heritage & Sefi, 1992) and laughter (Beach & Prickett, 2017; Glenn, 2003).

In the limited literature on explicit resistance to clinician advice (Bloch & Antaki, 2022), it tends to be related to territories of knowledge and epistemic claims (Heritage, 2012). Healthcare professionals often must decide how much to tell patients, how much to assume they know and how much to assume about patients’ life circumstances (Heritage, 2013). Getting this balance wrong can lead to resistance (Heritage, 2013), which can be displayed when patients respond by detailing their personal life experience, exerting their epistemic stance (White & Stubbe, 2023). When patients orient to primary access to their experience and display epistemic stance, they can also orient to their deontic rights (Ekberg & LeCouteur, 2015). Deontic rights relate to decisions on what should or should not be done in the future (Stevanovic & Perakyla, 2012). Weight loss advice can be a display of deontic rights over patients’ future behavior. In other advice contexts, resistance displays that orient to primary epistemic and deontic rights are difficult to manage, as clinicians are relatively unable to challenge patients’ personal experience (Bloch & Antaki, 2022; Ekberg & LeCouteur, 2015).

As yet, no published research has focused on how and where displays of explicit resistance to opportunistic weight loss advice manifest and are handled. Our prior research examined how clinicians can start talking about a patients’ weight and weight loss, noting that displaying delicacy can minimize resistance from patients, but this resistance was often implicit (Tremblett et al., 2022). In this current paper, we aim to examine how, in terms of specific words and phrases, clinicians offer opportunistic advice that patients respond to with explicit resistance displays. Understanding resistance in the context of opportunistic weight loss advice for people living with overweight and obesity is important to address clinicians’ concerns and assist them with the confidence to implement guidelines and recommendations.

## Method

### Context

Recordings were taken as part the “Brief intervention on weight loss” (BWeL) trial (full details can be read from Aveyard et al., 2016). GPs were asked to advise patients to change behavior to lose weight in their own words at the end of the consultation after managing the patients presenting concern (Lewis et al., 2013). The GPs managed the recording, turning the device on just before they initiated the intervention. See Tremblett et al. (2022) for further details on the data available for analysis. Ethical approval was granted by the NHS Research Ethics Service (reference: 13/SC/0028).

### Analysis

Analysis was led by MT, a qualitative researcher and conversation analyst with experience in analyzing healthcare encounters. For this paper, analysis focused on displays of explicit resistance by patients, with examination of what happened before these displays and GPs’ responses to them. Our prior publication has examined the GPs’ delicate approach to giving weight loss advice (Tremblett et al., 2022). Of the 237 recordings available, MT identified 45 instances of responses from patients that were displaying explicit resistance and these were transcribed using Jeffersonian conventions (Hepburn & Bolden, 2017). Explicit resistance was identified when a patient problematized the GPs’ project of providing advice, by showing that in some way it is not relevant or is redundant (Heritage & Sefi, 1992). We will show that this was done by patients displaying that the advice was known, or by appealing to factors that make the advice not suitable for that patient. As such, the patients were not cooperating with how they were positioned in the GPs’ turns at talk (e.g., that it was relevant or necessary to give advice on weight loss to the patients; Huma et al. (2023)).

Conversation analysis was then conducted on all instances of explicit resistance. Illustrations of the typical patterns found in analysis are shown in the findings. Conversation analysis attends to the structural and sequential organization of talk, capturing details in interaction that are important to intersubjectivity, including grammar, tone, and overlap (Sidnell, 2010). MT’s analysis had a specific focus on: (a) what happened before patient displays of resistance, (b) the format of the resistance displays, and (c) the ways in which the GPs managed these. MT used the next turn proof procedure (NTPP), where the action of a turn is understood by the response to it (Sidnell, 2010) in analysis. Analysis was developed in data sessions between the authors, and through a local data session (Oxford Researchers for Conversation Analysis – ORCA).^1^ Extracts were grouped into collections during analysis. We have provided the number of extracts in the patient response collections. These numbers are an indication of “prevalence” given interactions are dynamic and as such drawing hard boundaries between phenomena can be arbitrary. Pseudonymised excerpts are presented to illustrate and exemplify our findings.

## Findings

Resistance was not straightforward, with patients aligning to some elements of the GPs’ initiating actions, whilst resisting some elements of the GPs’ project that would make the advice relevant for the patient. We found patients displayed explicit resistance following clinician advice that was framed as a question (for example, “did you know if you were to lose weight it would help with your risk factors?”), or when the advice described how to lose weight through changes to diet or physical activity. The first section of analysis provides examples of these two precursors to displays of explicit resistance and the second examines the subsequent trajectory of the resistance and what happens next. The analysis demonstrates the role of epistemic territory and asymmetry in this resistance. Our analysis highlights the asymmetry of doctors (potentially) not knowing patients’ awareness of weight loss benefits, or what patients may have done or be doing to lose weight. Finally, we show how GPs managed these resistance displays.

### The precursors to explicit resistance

We describe the precursors to explicit resistance, with the format of the explicit resistance explored in the next section. In Extract 1, we show an example of the type of question that occurred before the explicit resistance displays. We join the extract as the audio recorder is turned on and the GP starts to talk to the patient about their weight. This was done once they had dealt with the “main business” of the consultation.

**Figure:**
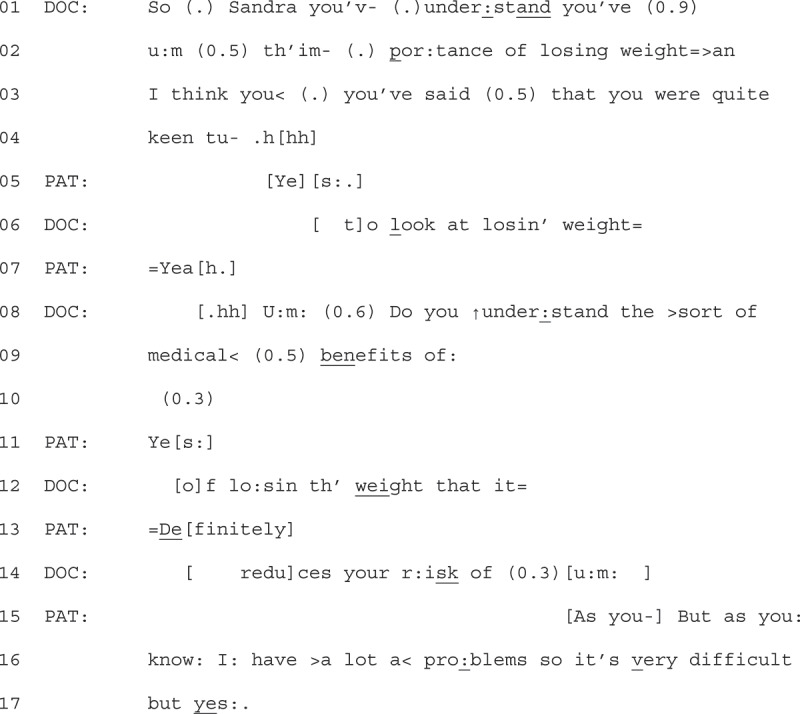
Extract 1 - 06-03-21

Here we see a GP start by using a Yes-No interrogative (YNI) to frame their talk about weight as a question for the patient to respond to. The GP prefaces the question, using a sequence-initiating “so” (Bolden, 2009), addressing the patient with their name (Sandra, line 1) which marks the relevancy for the patient to respond at the next transition relevance place (TRP). The GP continues their turn with talk that features a number of restarts and pauses (lines 01–04), which may orient to the delicacy of talking to the patient about their weight (Tremblett et al., 2022).

The GP’s prefacing on line 01 to 06 receives continuers from the patient (line 05 + 07) that align with the progressivity of the GP’s talk (Stivers, 2008). These patient responses signal a “go ahead” for the GP to continue discussing weight and weight loss. The GP then launches the YNI (line 08, “do you understand”), which also begins to link weight loss to medical benefits and a reduction of risk. Although the GP’s turn is hearable as grammatically incomplete on line 09 (“medical benefits of:”), with a continuation on the “of” that works to retain the turn at talk, the patient comes in with a clear “yes” at line 11. The GP treats this as an aligning continuer, moving forward with their turn, repeating “of” (line 12) to pick up where they had paused. The patient then upgrades their “yes” to “definitely” (line 13), responsive to the GP mentioning the benefits of losing weight. This upgraded affirmation appears to lead to a halt in the GPs progressivity. The GP abandons their turn, with a pause (0.3) followed by an “um” (line 14). The patient takes the opportunity to interject and remind the GP they already know some of the likely problems in overlap with the GP’s “um” (line 15). In this way, whilst the patient initially aligns with the GPs action of talking about weight (lines 05 + 07), they begin to display some resistance to the GP continuing their project of talking about the benefits of weight loss, only conditionally accepting the content of what the GP states with “but yes” on line 17. We will return to this extract to analyze the format of this patient’s resistance displays in the next section.

We now examine the type of diet and exercise advice that led to some displays of resistance. We join Extract 2 (on prior page) as the GP transitions to talking about weight, after turning on the audio recorder and stating the patient’s randomization number.

**Figure:**
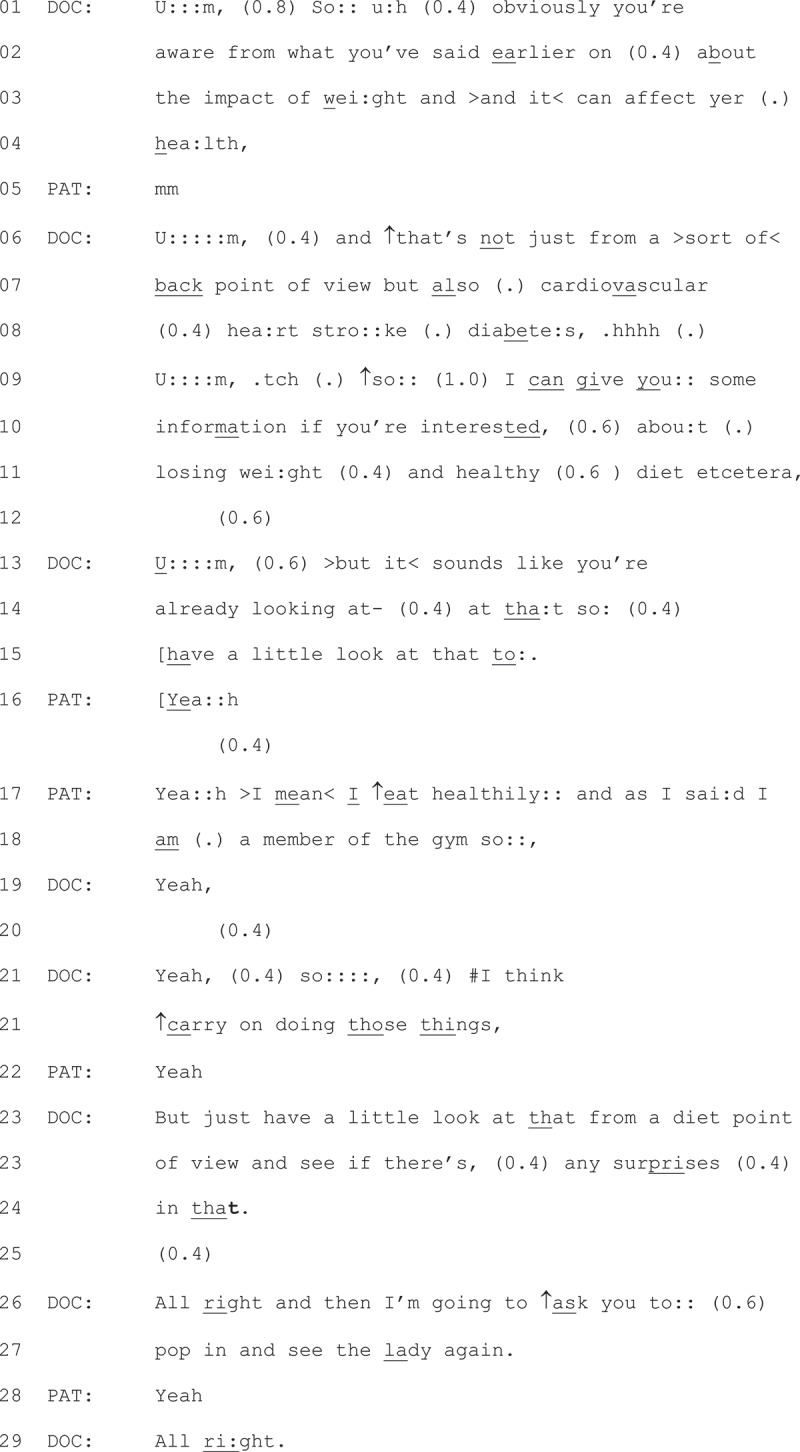
Extract 2- 20-02-01

The GP starts with a sequence-initiating “so” (line 01) (Bolden, 2009). In the pre-sequence that follows, the GP states “obviously you’re aware” (line 01). The use of “obviously” marks what the GP is stating as self-evident, and may work to manage potential/anticipated resistance (Hepburn & Potter, 2011). The GP then references something that the patient has already said, which works to personalize the talk to the patient (Tremblett et al., 2022). At this point, the patient responds with a minimal “mm” (line 05; Heritage and Sefi (1992)), which the GP treats as an aligning continuer).

With this continuer, the GP tailors their talk about weight loss benefits to the patient by referencing an existing problem (line 07 “back point of view”), before listing health conditions the patient may develop (lines 07 + 08). The GP moves to provide the upshot of their talk with a “so” prefacing (line 09, Raymond (2004)), before offering the patient information on how to lose weight and maintain a healthy diet. The design of this offer of information, links losing weight directly to a healthy diet, making an implicit suggestion that the patient requires advice on maintaining a healthy diet. The patient does not immediately respond to this offer (line 12). This lack of response leads to the GP repairing their offer (“but it sounds like you’re already”, line 13 + 14), marking that their original offer of information may have been misplaced. After what may have been an offer of an information leaflet (line 15, “have a look at this” – participating GPs had a healthy living booklet that they could choose to give patients), the patient responds. First, the patient responds with ‘yeah’s in overlap with the offer of an information leaflet (line 16), which could be marking some passive resistance (Bergen, 2020) by agreeing with the GPs suggestion (line 13 + 14) that they are already doing this behavior. The potential passive resistance then progresses to explicit resistance (line 17 + 18). The patient responds in a way that resists the advice giving, by asserting its redundancy, stating they already eat healthily and go to the gym. Therefore, the precursors to explicit resistance tread on patients’ knowledge, either about what health behaviors are or what they do to maintain their health.

### Format of explicit resistance in the consultations

As may be evident from the first two extracts, displays of explicit resistance were not straightforward. Alongside aligning responses to the GPs’ project of giving patients advice about weight loss, the patients made displays that demonstrated some trouble with the version of reality proposed in the GPs’ project (Huma et al., 2023). We have named these as displays of explicit resistance in contrast to passive resistance, however they could also be considered as implicit resistance displays (Riou et al., 2020), as they are implicitly problematizing the GPs’ project of providing advice, by showing that in some way it is not relevant or is redundant (Heritage & Sefi, 1992).

#### Resistance to questions

When GPs framed weight-loss talk as a question, resistance displays often targeted the content as *known* and *redundant*. We group five extracts in this collection. We can see an example of this by returning to Extract 1.

**1:**
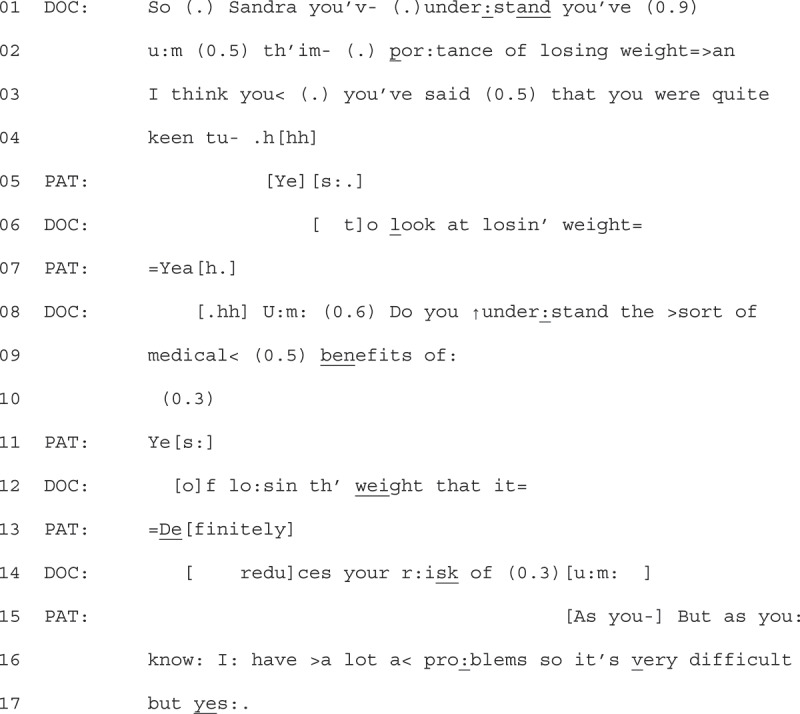
Extract 1 - 06-03-21

After initially aligning with the GP’s project to talk about weight (line 05 + 07), the patient then responds to the GP’s YNI (line 08–13) to display that the information is known. The patient does this by first responding with “yes” (line 11), providing an affirmation that they do understand. The placement of this “yes” is of note, as it particularly marks the known quality of the information, with confirmation made before the question is complete.

This knowledge display is further upgraded with “definitely” (line 13), shortly after the GP could have grammatically completed their question (after “weight”, line 12). These early confirmatory responses also decline to conform to the underlying action of the GP’s turn (information giving). We can see that the underlying action of the GP’s turn is information giving, rather than just a YNI, due to the post-expansion to the question which would otherwise be hearable as complete (after “weight”, line 12). The patient’s turn displays this information as redundant by closing the need for the post-expansion, preventing the progressivity of the GP’s turn to explain what potential risks weight loss would reduce. Instead, the patient further displays resistance to the action of GP’s turn by explaining how, as the GP should know, they have difficulties which make losing weight hard (lines 15, 16 + 17).

Extract 3 (below) shows explicit resistance to the GPs’ question design similar to that seen in Extract 1. We join the extract at the end of the consultation, after the GP has turned on the audio recorder and read the patients randomization number.

**1:**
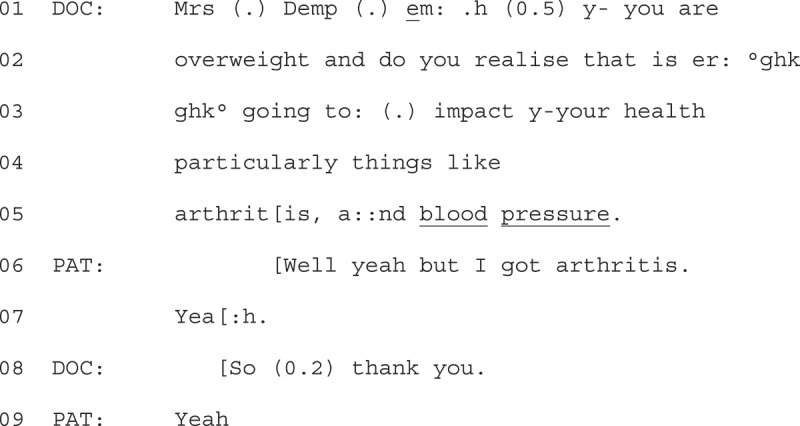
Extract 3- 05-01-04

The GP addresses the patient using their name and asserts that they are overweight (line 01 + 02), before formatting the advice that it is bad for their health as a YNI (“do you realize”, line 02). The GP then goes on to state that it will impact on the patients’ health, pinpointing arthritis and blood pressure as particular areas for concern (“particularly things like”, line 04). At this point, the patient starts to respond in overlap (line 06).

The overlap occurs after the GP mentions arthritis. The response is “well” prefaced (line 06), forecasting that it will be dis-preferred and non-straightforward (Heritage, 2015). The patient then demonstrates agreement that the information is known (“yeah”, line 06), before targeting the first health problem the GP mentions as already something the patient has (“I got arthritis”, line 06). In this way, the patient displays that this information is both known and redundant.

#### Resistance to diet and physical activity advice

When GPs gave some direction on how to lose weight through changes to diet or physical activity, explicit resistance was often displayed through claims to situational factors (“it’s been a rollercoaster in the past two months”), fixed physical states (“I can’t walk very far”) and assertions of previous efforts made to lose weight (“I’ve tried all the diets”). In the collections, we grouped four displays of resistance as using situational factors, 16 that used physical factors, and 20 that asserted previous efforts to lose weight. These numbers are used illustratively here, as patients sometimes drew on a range of factors to resist. Overall, patients were asserting epistemic claims that the GP “could not” or “did not” know.

##### Situational factors

Extract 4 (see next page) is an example of a patient displaying resistance through a claim to situational factors. We join the consultation after the GP has already stated that the patient is “carrying a lot of weight” and explained the health risks associated with “carrying lots of weight”. Up to this point, the patient has been responding with minimal continuers (e.g., “yeahs”).

**1:**
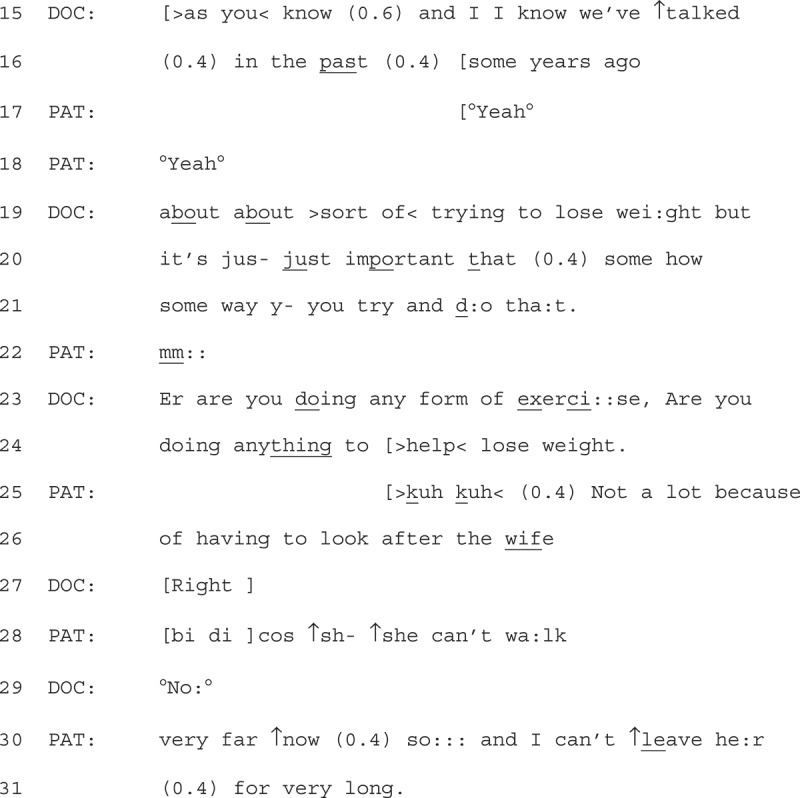
Extract 4 22-2-25

The GP then personalizes the talk to the patient by prefacing the advice, stating it is something they have already discussed “some years ago” (lines 15 + 16 (Tremblett et al., 2022)). The patient responds with minimal continuers (“yeah”, lines 17 + 18 (Heritage & Sefi, 1992)). The GP then continues their turn, displaying some delicacy (repetition of “about,” minimization using “sort of” (Tremblett et al., 2022)) before mentioning losing weight. The turn design places weight loss as a priority for the patient by stating it is “important” (line 20), and using the nonspecific, yet idiomatic “some how some way” (lines 20–21). This suggests that the patient should use any available method to lose weight. The advice receives a minimal response from the patient (line 22 “mm”), potentially forecasting some incipient resistance (Koenig, 2011).

The GP then states “are you doing any form of exercise, are you doing anything to lose weight” (line 23). The structure of these two questions, first a question about exercise, then a question on if they are doing anything to lose weight, ties exercise as a solution to lose weight. This question design also guides the response to focus on exercise (Pomerantz, 1988) and invites an account of what exercise they might be doing (Jefferson, 1989). The patient responds by accounting for why they are not doing exercise, and as such resists the implicit advice that exercise is a means for them to lose weight, without resisting the fact that they should be trying to lose weight. The patient discusses their personal situation, explaining that they have to look after “the wife” (line 25 + 26), before clarifying that she can’t walk (line 28), implicitly suggesting that this also limits his mobility. This implicit suggestion is expanded further with the continued turn taken by the patient (line 30 + 31), when the patient states he can’t leave her for very long (which may be required if he was doing exercise). By providing this account, the patient treats the GP as not already having access to this information.

##### Physical factors

Patients displayed resistance through claims to physical factors, as shown in Extract 5 (see previous page). We join Extract 5 40 seconds into the recording, after the doctor has talked about the patients’ health issues, how weight loss would help, that diet and exercise is important and that the practice nurse could help.

**1:**
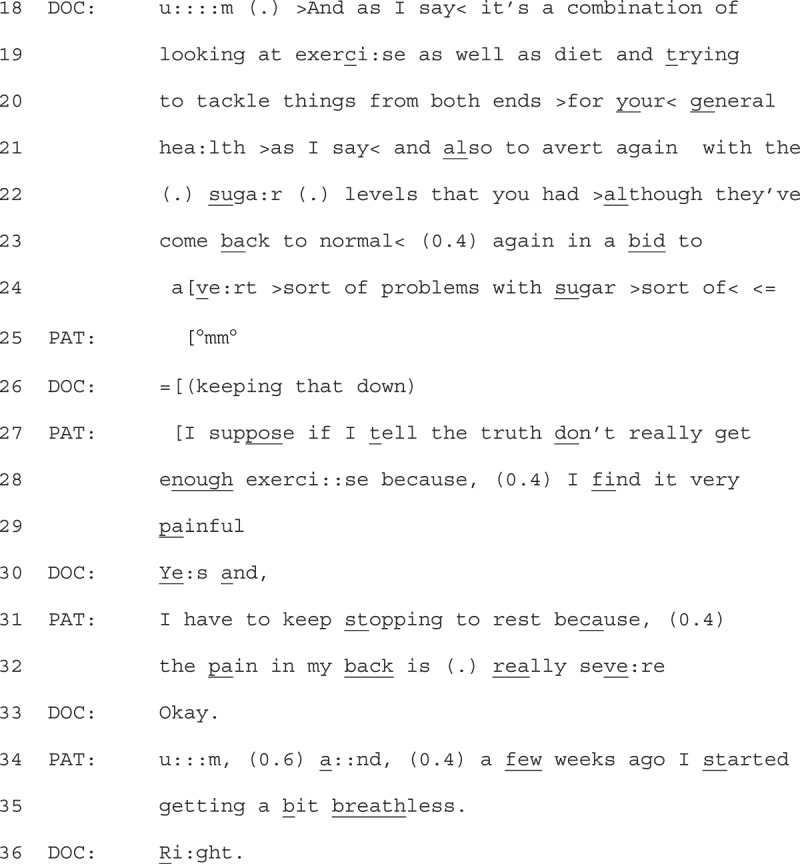
Extract 5 - 38-1-35 (40 seconds into the recording)

To this point the patient has responded minimally. The GP then returns to provide some advice on the method that the patient could follow to lose weight, stating “it’s a combination of … exercise as well as diet” (line 18). A benefit for general health is used to qualify this advice (lines 20 + 21), and the advice is personalized as helpful for one of the patient’s health issues (“avert again with the sugar levels”, lines 21 + 22). The GP’s turn then is designed to mitigate some potential resistance from the patient (e.g., that their sugar levels are fine now), by drawing on the need to consider that diet and exercise would help prevent future problems with sugar (line 24).

The patient responds to the GP with a turn that is designed like a confessional disclosure, prefacing their account with “I suppose if I tell the truth” (line 27) framing what they go on to say as information that the GP does not already know. This preface also treats the statement they go on to give, that “they don’t really get enough exercise” as a medical misdeed (Bergen & Stivers, 2013), demonstrating some alignment with the notion that they should be doing more to lose weight as proposed by the GP. They then use physical factors (back pain, line 31 + 32) to account for this misdeed, which works to resist the advice to do more exercise. The detailed account, and extreme case formulation of the back pain (Pomerantz, 1986) “really severe” (line 32), treats the GP as not having access to this knowledge already. By providing detail of a recent physical factor (“getting a bit breathless”, line 35), the patient further emphasizes this information as “new” and something that the GP is likely unaware of.

##### Asserting previous efforts

Patients would also resist advice by asserting their previous efforts to lose weight, as shown in Extract 6 (see previous page). The extract starts after consultation has finished, and the GP has turned on the audio recorder.

**1:**
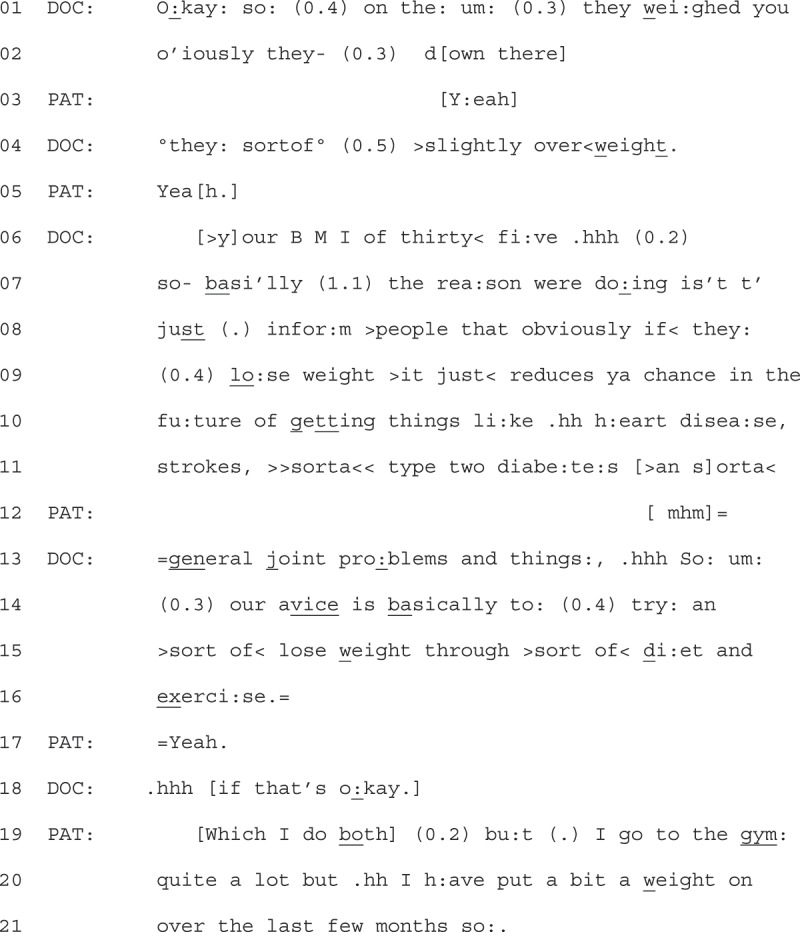
Extract 6 - 06-04-14

Here we see the GP talking to the patient about weight loss in a similar structure to the other extracts. They preface advice delivery by referencing what has already happened to the patient (they have been weighed, line 01 + 02), and then provide an appraisal of the patient’s weight (“slightly overweight” line 04). The GP’s talk contains delicacy features (e.g., hesitation (Tremblett et al., 2022)). In response, the patient states “yeah” (line 05), a minimal continuer which aligns with the progression of the GPs turn (Stivers, 2008). The GP continues their project on line 06, stating the patient’s BMI (body mass index), which is then followed by the GP stating “so basically” (line 07), tying the patients BMI as an account for what is to come (e.g., weight loss advice).

The GP explains that losing weight is good for “people” for a range of listed health reasons (lines 10–13), before providing the final “upshot” of their advice, marked by the “so” on line 13 (Raymond, 2004). After this so, and some marks of disfluency, the GP states that the advice is to lose weight through “diet and exercise” (line 15 + 16). Throughout this advice delivery, the GP displays delicacy, stating that this advice is for “people” in general (line 08, rather than this specific patient), avoiding personal pronouns and pausing which marks some hesitation (Tremblett et al., 2022). The patient receipts this advice with a short “yeah” (line 17), which is marked with a final intonation, suggesting alignment in receipt of the information. The patient then moves to continue their turn qualifying their receipt by stating “which I do both” (line 19). In this way, the patient implies that the GP’s advice is redundant. They further assert their prior effort to lose weight via exercise by stating that they have been “going to the gym quite a lot” (line 19), before using a contrastive “but” to state they have “put a bit of weight on” (line 20). After providing the time frame of this weight gain (“over the past few months”), the patient finishes their turn on “so:” (line 21). This “so” works to underscore the redundancy of the advice, leaving the recipient to infer the upshot of the fact that the patient has been exercising and is still gaining weight (Raymond, 2004).

### How GPs respond to explicit resistance

GPs would often respond to patients’ displays of explicit resistance by acknowledging the patient’s perspective, but rarely worked to overcome the resistance. Instead, progressivity was maintained when GPs minimized the action of their talk, and offered future support, such as another appointment that focused on weight-loss (moving the talk back into the clinician’s epistemic domain). An example of this is found by returning to Extract 1 (see previous page).

**1:**
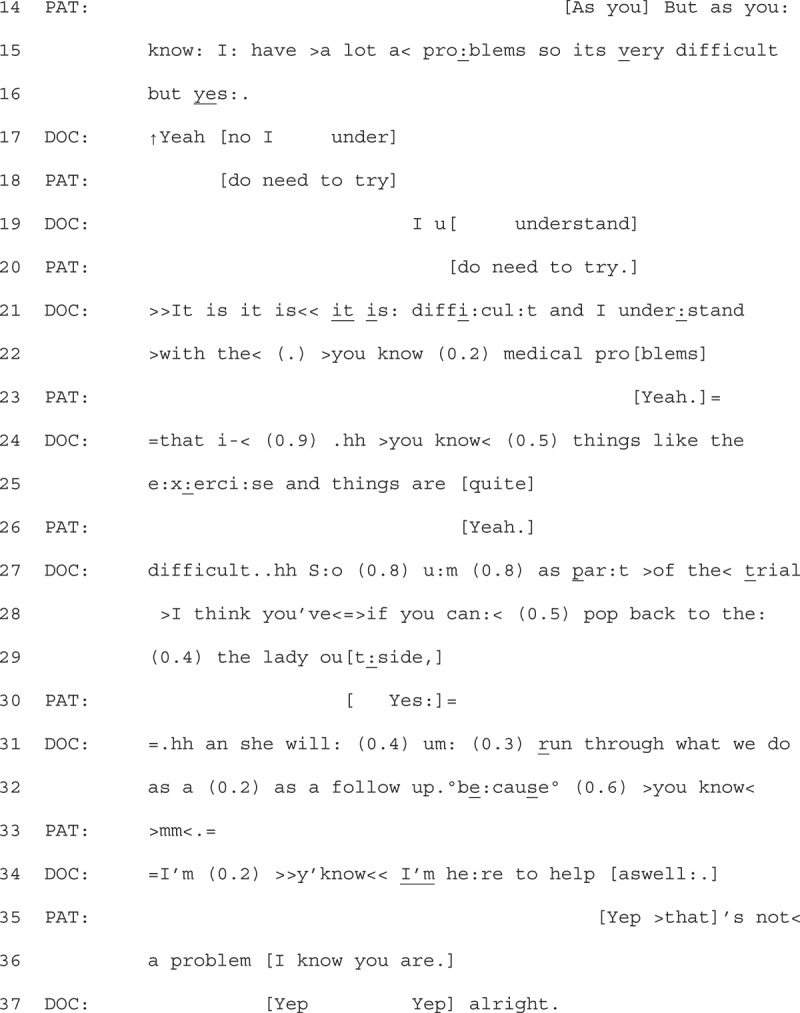
Extract 1 06-03-21

Following the patient claiming access to the GP’s knowledge (“as you know”, lines 14 + 15) of her “problems” that make weight loss “very difficult” (line 15), the GP responds by affiliating with the patient’s assertion. They do this by first stating “I understand” (line 17 + 19), overlapping the patients’ statement that they “do need to try” (line 18 + 20).

After the overlapping turns between the GP and patient, the GP expands on their turn to affiliate further, restating that they understand, using the patient’s words (e.g., “it is difficult” (line 21), “medical problems” (line 22)). They further display understanding by pinpointing what becomes difficult for the patient (“exercise” line 25). Throughout this extended turn, the GP demonstrates delicacy through perturbations (Silverman & Peräkylä, 1990). The GP then transitions to close the interaction, asking the patient to go back and see a researcher in reception (lines 27–32). Whilst outlining the next steps for the patient, they suggest that there may be a “follow up”, but trails off with the explanation (quietly stating “because”, followed by a pause “(0.6)”, and “you know”). They then hesitantly, seen with pauses (0.2) and restarts (I’m), assert and offer that they are available to help too.

### Accepted opportunistic advice

In Extract 7, we provide a brief example of a GP providing opportunistic advice to patient that is not responded to with explicit resistance. We have included this example to demonstrate that often, when GPs did not display a claim of what they thought the patients know about weight loss or what they might be doing to lose weight, displays of explicit resistance were not found. We join the extract as the GP at turned on the audio recorder at the end of the consultation.

**1:**
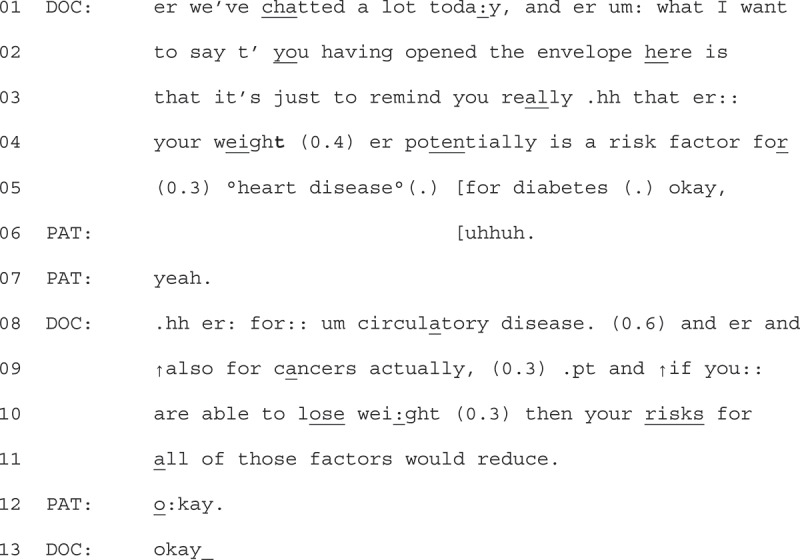
Extract 7 07-02-07

The GP starts by referencing back to what has already happened in the consultation on line 01 (Tremblett et al., 2022), then moves to frame their upcoming advice as a reminder (line 03). Prefacing their advice as a reminder works to display a stance toward the patient’s understanding of the benefits of weight loss. They then list the associated health risks of increased weight (lines 05, 08 + 09), which works to account for the advice they go on to provide (that if the patient loses weight their risks reduce, lines 09,10,11). The patient responds in an aligning manner with “okay” (line 12) (Stivers, 2008). Mindful of space, we have not provided the full interaction, but later in the discussion the patient closes by thanking the GP.

## Discussion

In this paper, we have unpicked the precursors to, and basis of, displays of explicit resistance when GPs provide opportunistic weight loss advice to patients living with obesity. Overall, we have demonstrated that weight is not inherently a resistible topic, rather resistance to opportunistic advice on weight loss relates to epistemic claims and territory. There were relatively few displays of explicit resistance across the recordings, and when they occurred, they were not straightforward. The rarity and nuance in the resistance displays may be related to interlocutors orientation to the asymmetrical roles of doctors and patients in healthcare encounters, with patients repeatedly found to avoid, or be very cautious about, overtly disagreeing with doctors (Pilnick & Dingwall, 2011). Thus, the lack of explicit resistance displays in general may not reflect anything particular about the topic of weight, weight loss, or opportunistic advice.

When explicit resistance did occur in our data, it often was preceded by GPs asking patients about their knowledge of the benefits of weight loss, or when they gave patients general advice on losing weight through changes to their diet or physical activity. Displays of explicit resistance tended to be based on claims relating to the patient’s personal experience, which effectively made the advice redundant. Responses from GPs tended to accept the patients’ resistance and did not pursue further weight loss advice apart from to offer future support.

Most prior research has focused on patients resisting medical advice despite having sought it in the first place (Bloch & Antaki, 2022; Ekberg & LeCouteur, 2015). Other researchers, examining opportunistic offers of referrals to weight management services for obesity, have found less pronounced interactional trouble if clinicians accept refusals rather than seeking to overturn them (Albury et al., 2022). However, recipients of an offer have refusal as a clear response option, which contrasts to responding with acceptance. Advice is a different action to which recipients have additional contingencies to manage in their response, depending on how the advice is formatted. For example, if oriented to as information it can be acknowledged in varying degrees (marked versus unmarked) which may convey acceptance or rejection, or redundancy (e.g., when assertions of knowledge are made (Heritage and Sefi, 1992) as we found in the data).

Displays of explicit resistance in opportunistic conversations about weight were often related to epistemic territory. Precursors to resistance targeted the patient’s epistemic grounds, positioning them as having less knowledge than the GP (Heritage, 2012). Querying a patient’s knowledge on weight loss benefits, whilst demonstrating a reticence to assume what the patient might know, also may implicitly suggest that they do not already hold this knowledge. Equally telling patients to make changes to their diet or physical activity may also imply they have a lack of knowledge and have not taken action on their weight. However, positioning patients as less knowledgeable has the potential to become unstuck in topics like weight, when weight management is often viewed and understood as managed by individuals, rather than clinicians (Busetto et al., 2022).

Patients made claims to knowledge about themselves when making displays of explicit resistance. These claims worked to redress the epistemic balance between the patient and GP, by positioning the GP as less knowledgeable about the patient’s personal activities (such as their gym membership or previous diet attempts). In this way, these explicit claims to personal experience effectively made the action of the prior turn (e.g., to advise patients to do more exercise) redundant.

Finding that most of the explicit resistance displays in the opportunistic weight loss advice conversations relates to epistemic territory reflects other findings on explicit resistance. Bloch and Antaki (2022) found that one of the ways that callers to a Parkinson helpline could resist was through personal epistemic claims. Equally, Ekberg and LeCouteur (2015) demonstrated that clients receiving therapy would also resist by using personal epistemic claims. These claims to personal knowledge assert the patients’ epistemic stance and work to claim the right to future courses of action (e.g., to refuse to go away and try to do more exercise because they know it is not physically possible; Ekberg & LeCouteur, 2015). Similarly to Bloch and Antaki (2022), we found that GPs would work to move the conversation forward after patients made displays of resistance based on personal experience, by accepting the patient’s claims and not challenging them.

Research on advice, and treatment recommendations, suggests that clinicians should work to overcome explicit resistance. This might be relevant in some medical arenas (e.g., when GPs are trying to avoid inappropriate antibiotic treatment, Stivers (2005)). However, when considering opportunistic weight loss discussions, this approach may not be warranted. First, we have shown that it was relatively rare for patients to explicitly resist the opportunistic advice. All patients agreed to participate in the trial (in which this data collection was nested) so had ostensibly agreed to talk about weight. However, they could still explicitly resist the ways in which weight was discussed. Accepting the patients’ resistance may be a way to demonstrate respect, and in other weight-related conversations acceptance of resistance supported smooth consultations and prevented escalation of resistance (Albury et al., 2022). Second, resistance tended to follow specific types of turns from the GPs. As such, rather than focus on overcoming resistance, it may be more prudent to focus on avoiding resistance (e.g., eschewing presuppositions about what the patient does or does not know, or do, about their weight). Managing weight is a long-term project for patients and clinicians. Maintaining positive interactions with patients and focusing on building collaborative relationships will likely be helpful in this long-term project.

Future research could examine if and how these explicit resistance displays translate into patient behavior. Although we did collect patient weight loss data, the small number of instances of explicit resistance precluded statistical analysis of their relationship with weight outcomes. The strength of the data on which the current analysis is based is that it captured multiple instances of opportunistic advice on the same topic. We have demonstrated that, by developing links with large trials, conversation analysts can examine consultations that are likely to reflect “usual” practice, capturing instances of things like opportunistic behavior change advice that may be difficult to access otherwise.

Existing literature suggests that weight is a difficult and sensitive topic to manage in the GP consultation. Our results show that difficulties made evident through resistance displays, are nuanced and related to *how* the topic of weight is discussed not discussion of weight per se. We found that resistance is associated with the sensitivity of managing epistemic claims and territory between speakers, and how claims to knowledge are asserted and managed. We have previously shown that if clinicians orient to talking about weight as a sensitive topic by using delicacy features, they can minimize resistance from patients (Tremblett et al., 2022). Explicit resistance can be avoided in many cases by a) not asking patients if they know that weight loss is beneficial, and b) not telling patients to change their diet or physical activity without knowing about the patient’s current behaviors.

## Note

1. See Stevanovic and Weiste (2017) for data sessions’ role in CA.
